# The Rise and Fall of MAIT Cells with Age

**DOI:** 10.1111/sji.12237

**Published:** 2014-11-26

**Authors:** L J Walker, H Tharmalingam, P Klenerman

**Affiliations:** * Newcastle University, Institute of Cellular MedicineNewcastle upn Tyne, Tyne And Wear, UK; † Nuffield Department of Medicine, University of OxfordOxford, Oxfordshire, UK

To the Editor

We read with interest the article by Novak *et al*. recently published in the Scandinavian Journal of Immunology and write in support of their data.

The authors describe the profound changes with age of peripheral circulating mucosal associated invariant T (MAIT) cells in a cohort of patients whose samples were obtained post-clinical analysis 1. We conducted a similar study of a cohort of 160 patients aged <1–90 years (mean 41 years) a number of years ago of patients attending the John Radcliffe Hospital in Oxford, UK where we performed whole blood antibody staining of samples post-clinical analysis. Unlike Novak *et al*., we did not know the patient background and were unable to exclude patients with infective or inflammatory conditions, however, similarities between the data sets and also this recently published paper 2 are striking.

**Figure 1:**
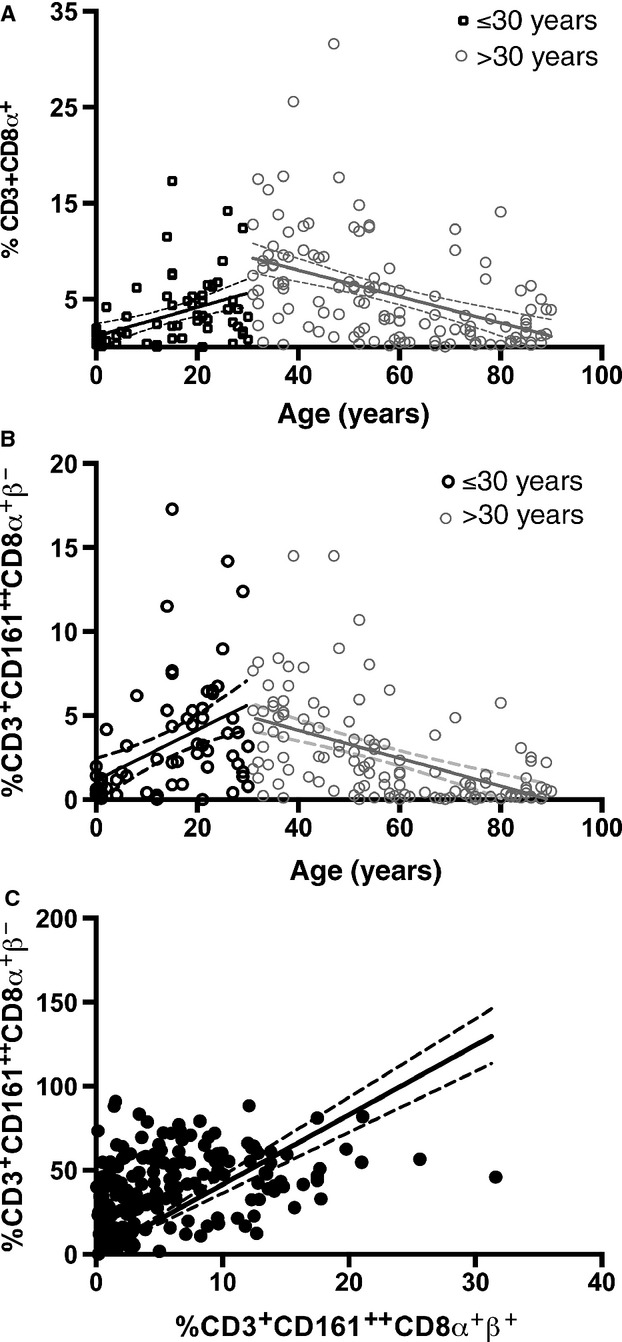
Variation with age of peripheral CD3+CD161++CD8*α*+ T cells. (A) Correlation between age and the size of the CD3+CD161++CD8*α*+*β*+ subset as a proportion of CD3+CD8*α*+*β*+ T cells in patients < or ≥30 years old. (<30 years *r* = 0.4651, *P* < 0.0001; ≥30 years *r* = −0.5171, *P* < 0.0001 Spearman's rank). (B) Correlation between age (years) and the size of the CD3+CD161++CD8*α*+*β*- subset as a proportion of CD3+CD161++CD8*α*+ T cells < or ≥30 years old (<30 years *r* = 0.5358 *P* < 0.0001; ≥30 years *r* = −0.5878 *P* < 0.0001 Spearman's rank). (C) Relationship between CD161++CD8*α*+*β*+ and CD161++CD8*α*+*β*- subsets as a proportion of CD3+ cells (*r*^2^ = 0.1151, *P*<0.0001 Linear regression).

Novak *et al*. describe the MAIT cell population as CD3+CD161++V*α*7.2+ cells; however, the naïve CD3+CD161++ population in cord blood has polyclonal T cell receptor usage and mature V*α*7.2+ and V*α*7.2- CD161++ T cell subsets share the same distinctive phenotype and function of the MAIT population 3,4. Our study was conducted before wider availability of the V*α*7.2 antibody and describes the CD3+CD161++CD8*α*+ population that includes both V*α*7.2+ and V*α*7.2- cells, representing about 90% of the MAIT cell population, with the remainder predominantly double negative CD4-CD8- cells (DN) 3. We found a significant positive correlation between peripheral blood CD3+CD161++CD8*α*+ cell frequency and age up 30 years (*r* = 0.4651, *P* < 0.0001) and subsequently a significant negative correlation between MAIT cell frequency and age in those patients ≥30 years (*r *= −0.5171, *P *< 0.0001) (Fig.1A).

CD8 can be expressed as both a CD8*αβ* heterodimer or a CD8*αα* homodimer and the CD3+CD161++CD8*α*+/MAIT population further subdivides into CD8*α*+*β* ^-/low^ (CD8*αβ* and CD8*αα* expressing) and CD8*α*+*β*- (CD8*αα* single positive) subsets 3,5,6. On further analysis, here, we describe the CD161++CD8*α*+CD8*β*- subset to vary in a similar pattern with age to the overall CD3+CD161++CD8*α*+ subset (<30 years *r* = 0.45358 *P* < 0.0001; ≥ 30 years *r* = −0.5878 *P* < 0.0001) (Fig.1B) and a significant positive correlation between the size of the CD161++CD8*α*+*β*+ and the CD161++CD8*α*+*β*- as a proportion of CD3+ cells (Fig.1C). Novak *et al*. describe a fall in CD8*α*+ MAIT cells and increase in proportion of DN MAITs with increasing age. Our own previously published data would indicate CD161++CD8*αα* MAIT cells to be derived from CD161++CD8*αβ* cells; however, the origin of the DN subset is not known 3. Our data would indicate that the proportion of CD161++CD8*αα* subset remains in a steady state as a proportion of CD161++CD8*α*+ MAIT population overall, regardless of possible progression to DN status.

Our data strongly support the conclusions of the authors that further work involving MAIT cells should ensure careful age and sex-matched controls are used to allow for appropriate interpretation of data suggesting changes in MAIT cell frequency within particular disease states; uncontrolled studies of MAIT cells require cautious interpretation.

Yours Sincerely

Dr Lucy Walker

Dr Hannah Tharmalingham

Professor Paul Klenerman
